# Sustained delivery of neurotrophic factors to treat spinal cord injury

**DOI:** 10.1515/tnsci-2020-0200

**Published:** 2021-11-30

**Authors:** Aikeremujiang Muheremu, Li Shu, Jing Liang, Abudunaibi Aili, Kan Jiang

**Affiliations:** Department of Spine Surgery, Sixth Affiliated Hospital of Xinjiang Medical University, 39 Wuxing Nan Rd, Tianshan District, Urumqi, Xinjiang, 86830001, People’s Republic of China; Department of Orthopedics, Sixth Affiliated Hospital of Xinjiang Medical University, Urumqi, Xinjiang, 86830001, People’s Republic of China; Department of Laboratory Medicine, Sixth Affiliated Hospital of Xinjiang Medical University, 39, Wuxing Nan Rd, Tianshan District, Urumqi, Xinjiang, 86830001, People’s Republic of China

**Keywords:** spinal cord injury, neurotrophic factors, sustained delivery, stem cells, viral vectors, tissue engineering, scaffolds

## Abstract

Acute spinal cord injury (SCI) is a devastating condition that results in tremendous physical and psychological harm and a series of socioeconomic problems. Although neurons in the spinal cord need neurotrophic factors for their survival and development to reestablish their connections with their original targets, endogenous neurotrophic factors are scarce and the sustainable delivery of exogeneous neurotrophic factors is challenging. The widely studied neurotrophic factors such as brain-derived neurotrophic factor, neurotrophin-3, nerve growth factor, ciliary neurotrophic factor, basic fibroblast growth factor, and glial cell-derived neurotrophic factor have a relatively short cycle that is not sufficient enough for functionally significant neural regeneration after SCI. In the past decades, scholars have tried a variety of cellular and viral vehicles as well as tissue engineering scaffolds to safely and sustainably deliver those necessary neurotrophic factors to the injury site, and achieved satisfactory neural repair and functional recovery on many occasions. Here, we review the neurotrophic factors that have been used in trials to treat SCI, and vehicles that were commonly used for their sustained delivery.

## Graphical abstract

**Figure:**
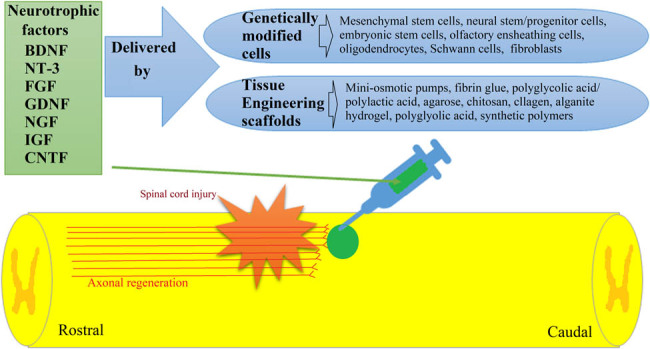

Acute spinal cord injury (SCI) is often caused by high-energy trauma such as traffic accidents, falls, physical injuries, stab, and gunshot wounds, and causes devastating motor, sensory and autonomic dysfunctions that often lead to patient morbidity and mortality [1,2]. With the industrialization of societies, increasing height of buildings, and popularity of private cars, the prevalence of vertebral fractures combined with SCI caused by falling and traffic accidents is steadily on the rise over the last decade. Those high-energy incidents are more prevalent in young male adults, who are the major workforce in construction, transportation, mining, and other industries, and the main breadwinner of their families [3]. Serious physical and psychological trauma, long-term high-cost rehabilitation treatment can be devastating to their families both economically and psychologically, possibly leading to serious socioeconomic problems. The direct life-long cost for the care and treatment of patients with SCI is about 1.1–4.6 million US dollars [4,5]. Therefore, the prevention, treatment, and rehabilitation of SCI is a topic that requires urgent attention and in-depth investigation.

## 1 Neurotrophic factors for SCI

After the incidence of SCI, neurons in the spinal cord need neurotrophic factors to guide their growth and development and reestablish their connections with their original target organs [6]. The deficiency of endogenous neurotrophic factors at the SCI site leads to axonal deformation and even neuronal apoptosis [7]. Neurotrophic factors are characterized as either endogenous or exogenous origins. Endogenous neurotrophic factors and their receptors are widely present in the spinal cord. However, except for neurotrophic factor-3, whose expression is relatively high in the early stage of spinal cord development, the amounts of other neurotrophic factors are often too low to exude a positive effect on neural regeneration in the spinal cord [8]. Geng et al. [9] measured that brain-derived neurotrophic factor (BDNF) increased 24 h after SCI and returned to normal level after 28 days. Tyrosine kinase receptor C (TrkC) protein decreased within 7 days after spinal cord transection and began to increase after 7 days, which was significantly accelerated in another 7 days. This was consistent with the changes of TrkC mRNA expression [10]. The mRNA expression of the p75 neurotrophic factor receptor fluctuated after SCI and was positively correlated with neuronal apoptosis, which could be a regulatory factor during the neuronal repair of SCI [11]. The upregulation of neurotrophic factors and their receptors after SCI is time-dependent and has varied positive effects on promoting spinal cord repair. However, due to their limited concentration at the site of injury, endogenous neurotrophic factors alone cannot maintain the effective therapeutic concentration for a sustained time. Therefore, adequate and sustained delivery of exogenous neurotrophic factors to the injury site is necessary to promote neural repair and functional recovery after SCI.

In the treatment of SCI, currently, the most commonly used exogenous neurotrophic factors include BDNF, neurotrophin-3 (NT-3), nerve growth factor (NGF), ciliary neurotrophic factor (CNTF), basic fibroblast growth factor (bFGF), insulin-like growth factor (IGF), and glial cell-derived neurotrophic factor (GDNF). They have been widely reported to promote the survival of nerve cells, stimulate the growth of axons, and promote functional recovery after SCI [12].

### 1.1 BDNF

BDNF binds to tyrosine kinase receptor B (TrkB), induces TrkB phosphorylation and activation of intracellular signaling pathways, reduces apoptosis of spinal cord motor neurons, and elevates the expression of 5-hydroxytryptamine at the injury site. It maintains and promotes the development, differentiation, and regeneration of sensory neurons, cholinergic neurons, dopaminergic neurons, and d-aminobutyric acid neurons [13]. According to Brock et al. [14], exogenous BDNF can reduce cortical atrophy, and significantly, increase the number of axons and myelin sheath formation at the injury site. BDNF can reduce and reverse the atrophy of injured neurons, and promote the regeneration and functional recovery of the rubrospinal tract after SCI. Han et al. [15] reported that sustained delivery of BDNF with collagen scaffolds to the injury site can significantly increase regeneration of axons in the corticospinal tract (CST) after SCI. Li et al. [16] injected the BDNF gene-modified bone marrow mesenchymal stem cells (MSCs) into the acute SCI site of adult SD rats, and observed increased survival rate of neurons at the injury site, indicating that BDNF can rescue the injured anterior horn neurons and promote the recovery of motor function under the injury level.

### 1.2 NT-3

NT3 is one of the most potent neurotrophic factors in neuronal regeneration and functional recovery after SCI [17]. In addition to maintaining the survival of sympathetic neurons, sensory neurons, basal forebrain cholinergic neurons, and motor neurons, NT3 can also support the differentiation of dopaminergic neurons and promote the sprouting of the lateral branches of the CST [18,19]. NT-3 receptor TrkC is mainly concentrated on axons. NT-3 from presynaptic neurons is needed for TrkC-dependent competitive dendrite morphogenesis in postsynaptic neurons [20]. NT-3 has been widely tested in animal experiments, and mostly delivered to the injured spinal cord and its adjacent regions shortly after the injury. Kadoya et al. [21] found significant improvement of spinal cord function after increasing the concentration of NT-3 and the surrounding microenvironment. Some studies have shown that NT-3 has a positive effect on the formation of the myelin sheath. When neurotrophic factor 3 is combined with other neurotrophic factors such as BDNF, the atrophy, and death of neurons in the rubrospinal tract can be further reduced [22].

### 1.3 CNTF

CNTF is an acidic protein with a relative molecular weight of 20,000–24,000 that promotes the survival of neurons, with a special protective effect on motor neurons. In the study of Cao et al. [23], the survival of grafted CNTF-OPCs increased fourfold compared with control-OPCs, and CNTF significantly increased the percentage of adenomatous polyposis coli oligodendrocytes (OLs) from grafted OPCs, which formed central myelin sheaths around the axons in the injured spinal cord, and significantly enhanced the recovery of hindlimb locomotor function, indicating that Schwann cells (SCs) transfected with CNTF gene can be used to bridge tissue defects during neural reconstruction after SCI.

### 1.4 Basic fibroblast growth factor

FGFs can promote the survival and regeneration of a variety of central and peripheral neurons both *in vivo* and *in vitro*. According to the different isoelectric characteristics, FGF can be divided into acidic FGF (aFGF) and basic FGF (bFGF). Because aFGF has no signal peptide, it cannot be synthesized and secreted from cells, and may play a nutritional role through internal secretion. bFGF can provide neurotrophic support to spinal cord neurons, and selectively promote axonal growth [24]. The possible working mechanism of bFGF is to inhibit apoptosis and c-fos gene expression at the injury site, stabilize calcium and magnesium ion levels and inhibit their toxicity, regulate the reaction of glial cells and reduce glial scar formation [25]. Goldshmit et al. [26] found that subcutaneous injection of fibroblast growth factor 2 in adult mice can reduce activation of microglia and macrophages and inhibit the inflammatory reaction. Reis et al. [27] used FGF-2 that was encapsulated into core–shell microfibers by coaxial electrospinning to treat rat SCI and found that it supported the survival and proliferation of PC12 cells *in vitro*, and increased the locomotor recovery of the animals 4 weeks after injury.

### 1.5 GDNF

GDNF belongs to the transforming growth factor b (TGF-b) superfamily. It has a strong neurotrophic effect on the motor, sensory, and dopaminergic neurons, and its therapeutic effect on central and peripheral nervous system injuries has been confirmed in previous studies [28]. Sharma [29] applied high concentrations (0.5 mg) of BDNF and GDNF after SCI and found that motor function recovery and inhibition of blood spinal cord barrier damage were significantly improved, neuronal apoptosis was inhibited and neural regeneration was promoted. When the lentivirus-mediated GDNF secreting cells were transplanted to the SCI site, the density of nerve fibers at the injured site was significantly increased, and the motor function was better restored [30], showing that lentivirus-mediated GDNF gene transfer can increase the secretion of GDNF for a prolonged time, promote the recovery of injured sensory and motor neurons and the regeneration of motor axons. Meanwhile, GDNF also causes axon trapping. Verhaagen et al. reported that, in the peripheral nervous system, although GDNF can promote the survival and outgrowth of motoneurons, locally elevated levels of GDNF could cause trapping of regenerating axons and the formation of nerve coils. They used lentiviral vectors to create gradients of GDNF in the sciatic nerve after ventral root avulsion and found that controlled expression of GDNF can be used to avoid motor axon trapping [31].

Single or a cocktail of exogenous neurotrophic factors can reach the spinal cord through the vein, abdominal cavity, muscle, and subcutaneous tissue injections. However, the concentration of neurotrophic factors in plasma decreases rapidly after administration through those routes. Due to the high molecular weight of neurotrophic factors, only a small portion of in plasma can pass through the blood spinal cord barrier to the injury site [32]. Although direct injection into the injury site or subarachnoid space could guarantee high concentrations of neurotrophic factors where they are needed, they could easily spread, and due to the short half-life cycle of neurotrophic factors, they cannot provide continuous neurotrophic support to the neurons [33]. In the treatment of SCI, it is vital to find economic and easily available methods to sustainably deliver various neurotrophic factors to the injury site to support neural regeneration and functional recovery. Precursor or stem cells transferred with vectors encoding the sequence of desired neurotrophic factors, recombinant proteins such as osmotic pumps, nanoparticles, viral vectors, as well as polymer scaffolds, were used to sustainably deliver the much needed neurotrophic support in the site of SCI.

## 2 Stem cells overexpressing neurotrophic factors

Stem cells have long been used to consistently secrete various desirable proteins after being genetically manipulated in regenerative medicine for a few decades. Stem cells have the ability of self-renewal and can differentiate into functional cells of specific tissues. They can be used as seed cells in various tissue regeneration and repair processes. Previous studies have shown that cell transplantation can be safely and effectively promote the recovery of motor, sensory or autonomic nerve function after SCI [34,35]. Transplanted cells can regulate immune function, produce various neurotrophic factors, and promote neural and vascular regeneration, which is closely related to functional recovery after SCI [36]. A variety of cells, including MSCs, neural stem cells (NSCs), embryonic stem cells (ESCs), olfactory ensheathing cells (OECs) as well as OLs have been used to repair SCI (Table 1). Together with various viral vectors (Table 2), different neurotrophic factors were successfully delivered in a sustained and controlled manner to promote neural regeneration after SCI.

**Table 1:** Cellular vehicles that were used to deliver neurotrophic factors in the treatment of SCI

Cellular vehicles	Animal	Injury	Site	Neurotrophic factors	Outcome	Reference
Schwann cells	Rat	Hemisection	T8	BDNF and NT-3	Improved axonal regeneration, no changes axonal myelination	[37]
Schwann cells	Rat	Complete transection	T9-T10	NT-3	Increased number of labeled interneurons and relay neurons	[38]
Schwann cells	Rat	Contusion	T10	NGF + BDNF	Enhanced BBB functional outcome	[39]
Schwann cells	Rat	Complete transection	T7	GDNF	Significantly increased axonal regeneration and myelination, no significant functional improvement	[40]
Schwann cells	Rat	Lateral hemisection	T8	GDNF	Increased myelination, reduced macrophage infiltration and cysts formation	[41]
Schwann cells	Rat	Complete transection	T10	GDNF	Increased axonal growth and myelination	[42]
Schwann cells	Rat	Hemisection	T11	GDNF	Accelerated synaptic growth and functional improvement	[43]
Schwann cells	Rat	Contusion	T10	GDNF	Increased cell survival and tissue sparing, better functional recovery	[44]
Schwann cells	Rat	Hemisection	T10	GDNF	Reduced the lesion cavity, astrocytic gliosis, and inflammatory responses at the graft-host boundaries, enhanced axonal regeneration	[45]
BDMS	Rat	Dorsal column transection	C3	BDNF	Improved host axonal growth and graft penetration, but little descending axonal penetration, and no significant improvement	[46]
BDMS	Rat	Dorsal hemisection	C4	BDNF	Improved axonal penetration in the injury site	[47]
BDMS	Rat	Complete transection	T8	BDNF	Improved survival of neurons but no significant difference in functional recovery	[48]
BDMS	Rat	Contusion	—	BDNF	Increased cellular survival and improved nerve function	[16]
NSCs	Rat	Hemisection		NT-3	Enhanced therapeutic results	[49]
NSCs	Rat	Complete transection	T10	BDNF	Improved NSCs survival and neuronaloglial cell differentiation, significant hindlimb locomotion, and sensory function recovery	[50]
NSCs	Rat	Complete transection	T8	BDNF, NT3 and NGF	Limited CST regeneration but significant functional improvement	[51]
NSCs + Amniotic epithelial cells	Rat	Contusion	T10	FGF-2	Improved neuronal cell survival and differentiation, better functional recovery	[52]
Neural stem/progenitor cells	Rat	Hemisection	T13	BDNF	Improved mechanical and thermal allodynia responses	[53]
Neural stem/progenitor cells	Rat	Contusion	T8	GDNF	No significant change in cellular and tissue survival, no significantly better functional recovery	[54]
Neural stem/progenitor cells	Rat	Contusion	T8	GDNF	Promoted neurite outgrowth, axonal regeneration and myelination, and improved locomotor recovery	[55]
Fibroblasts	Rat	Subcortical lesions	T7	BDNF	Improved survival of neurons, and increased motor and sensory axons	[56]
Fibroblasts	Rat	Dorsal hemisection	T7	BDNF	Increased axonal outgrowth and the density of SCs but no synapse formation	[57]
Fibroblasts	Rat	Lateral hemisection	C5 C6	BDNF + NT-3	No significant functional recovery despite significantly improved serotonergic innervation	[58]
Fibroblasts	Rhesus monkey	Lateral hemisection	C7	BDNF and NT-3	Significantly alleviated corticospinal neuronal atrophy over extended distances increased axonal growth	[14]
OECs	Rat	Complete transection	T8	GDNF	Increased nerve fiber regeneration and functional recovery	[59]
OECs	Rat	Dorsal column transection	C4	NT-3	Reduced lesion volume, and increased axonal regeneration, no clear functional recovery	[60]
OECs	Rat	Left dorsolateral transection	C4	NT-3	Extensive axonal sprouting and enhanced hindlimb recovery.	[61]
Mesenchymal precursor cells	Rat	Hemisection	T10	BDNF	Significantly improved remyelination and functional recovery	[62]
Mesenchymal precursor cells	Rat	Contusion	T7-8	BDNF + NT-3	Reduced cyst size, improved neuronal survival, and functional recovery	[63]
Mesenchymal precursor cells	Rat	Contusion	T9	NGF	Reduced cyst size, improved axonal regeneration, and functional recovery	[64]
Mesenchymal precursor cells	Rat	Dorsal hemisection	T9	BDNF	Improved CST neuron survival, CST sprouting, and functional recovery	[65]
MSCs	Rat	Transection	T9	NT-3	Improved motor outcomes and tissue continuity at the transection site despite no evidence of axon regeneration	[66]
Human umbilical cord blood mononuclear cells	Rat	Contusion	T8	VEGF and GDNF	Decreased cellular apoptosis. Increased axonal generation and myelination	[67]

Abbreviations: BDMS: bone marrow-derived mesenchymal stem cells; BDNF: brain-derived neurotrophic factor; NT-3: neurotrophin-3; NGF: nerve growth factor; GDNF: glial cell-derived neurotrophic factor; VEGF: vascular endothelial growth factor.

**Table 2:** Viral vectors that were used to deliver neurotrophic factors in the treatment of SCI

Viral vectors	Animal	Injury	Site	Neurotrophic factors	Outcome	Reference
Lentivirus	Rat	Dorsal hemisection	C2/C3	NT-3	Increased axonal density within cellular grafts, negative axodendritic synapses, and increased lesion size	[68]
Lentivirus	Mice	Clip compression	T9	BDNF	Increased myelination of infiltrating axons, modest improvements to the hindlimb function	[69]
Lentivirus	Rat	Clip compression	T9	bFGF	Increased neuronal survival, and inhibited autophagy in spinal cord lesions, and improved functional restoration	[70]
Lentivirus	Rat	Complete transection	T10	Netrin-1	Significantly improved motor and sensory functional recoveries	[71]
Adenovirus	Rat	Hemisection	T8	NT-3	Significant promotion of CST axonal growth when the NT-3 source. CST collaterals – need to make NT-3 available in close proximity to CST target axons	[72]
Adenovirus	Rat	Complete transection	T9-10	BDNF	Significantly increased glutamatergic and GABAergic neurotransmission	[73]
Adenovirus	Rat	Contusion	T9	FGF-1	Significant functional improvement	[74]
Adenovirus + SCs + hyrdogel	Rat	Hemisection	C5	BDNF	Increased axonal regeneration	[75]
Adenovirus	Rat	Hemisection	C2	BDNF	Robust axonal sprouting and improved functional recovery	[76]

Abbreviations: BDNF: brain-derived neurotrophic factor; FGF: fibroblast growth factor; NT-3: neurotrophin-3.

### 2.1 MSCs

MSCs are first isolated from bone marrow and now can be obtained from adipose tissue, placenta, as well as peripheral blood. MSCs can regulate immune response and secrete a variety of immunomodulatory and neurotrophic factors including interleukin-6 (IL-6), NGF, BDNF, GDNF, and the vascular endothelial growth factor (VEGF). MSCs have been widely used in the repair of SCI due to their easy isolation, proliferation, and low immunogenicity [77,78,79,80,81]. White et al. used mesenchymal progenitor cells injected via the tail vein of mice cervical SCI models 1 to 10 days after injury and found that those cells were evenly distributed in lungs, and may play as cellular target decoys to the immune system of mice SCI model, and reduce secondary injury to the spinal cord tissue [82]. When MSCs overexpressing TrkC and NT-3 were transplanted into the SCI site of adult rats, the motor evoked potential and hindlimb function were significantly improved after 8 weeks compared to nontransfected MSCs [83,84]. Those studies showed that genetically modified MSCs that can consistently secrete certain neurotrophic factors can be effective and safe tools to promote nerve regeneration and nerve function recovery.

### 2.2 NSCs

NSCs are undifferentiated cells that exist in specific parts of the central nervous system during development and adulthood. They can self-renew and differentiate into neurons, astrocytes, and OLs in the nervous system [85]. Reynolds et al. [86] successfully isolated NSCs from adult mouse striatum in 1992. NSCs can be isolated or induced from developing and adult neural tissues, ESCs, as well as induced pluripotent stem cells (IPSCs).

However, although cellular transplantation has shown great potential in the treatment of SCI, there are still many hurdles that limit its further application, the most important being the diffusion of stem cells after injection. Because of the dynamic cerebrospinal fluid in the spinal cord, it is difficult for NSCs to colonize the injury site. It could be an effective strategy to limit the proliferation of stem cells by transplanting stem cells into the injury site using tissue engineering scaffold materials as a method of sustained drug delivery [87].

## 3 Biomaterials for sustained neurotrophic factor delivery

Tissue engineering scaffolds and biomaterials can provide support and guidance for the regeneration of injured nerves and build a bridge for nerve regeneration in the injured area [88]. At the same time, it can be used as a carrier of neurotrophic factors or stem cells overexpressing various neurotrophic factors. Nerve scaffold materials combined with neurotrophic factors and stem cells form functional biomaterials that can be used to treat the SCI [89]. Nerve scaffolds can be composed of both synthetic and natural materials. Synthetic materials have the advantage of easy mass production and easy control of physical properties but they could have poor cell compatibility. The acidic environment after the degradation of synthetic materials may affect the survival and growth of cells. The main components of natural materials are extracellular matrices such as natural macromolecular proteins, collagen, and fibrin, and natural polymers such as hyaluronic acid and chitosan. Their main advantages are the convenient source and low immunogenicity. When the neurotrophic factors are imbedded in nerve scaffolds, they can effectively maintain their therapeutic concentration at the injury site by modulating the scaffold degradation and the kinetics of release, reducing the overall dosage, and avoiding side effects of multiple injections [90,91]. Mini-osmotic pumps, fibrin glue, polyglycolic acid/polylactic acid, agarose, chitosan, as well as the acellular spinal cord, were used successfully to sustainably deliver neurotrophic factors to the injury site and promote cellular survival, tissue sparing, axonal regeneration, and functional recovery (Table 3).

**Table 3:** Tissue engineering scaffolds that were used to deliver neurotrophic factors in the treatment of SCI

Tissue engineering grafts	Subjects	Injury	Site	Neurotrophic factors	Outcome	Reference
Mini-osmotic pump	Rat	Dorsal column and CST ablation	T9–T10	BDNF	Enhanced axonal sprouting rostral to graft	[92]
Mini-osmotic pump	Rat	Dorsolateral transection	C4	BDNF	Improved neuronal regeneration	[93]
Mini-osmotic pump	Rat	Contusion	T9	NT-3	No protective effect was found from the degeneration of ascending sensory axons	[94]
Mini-osmotic pump	Rat	Contusion	T10	FGF-2	Improved tissue protection and functional recovery	[95]
Mini-osmotic pump	Rat	Clip compression	T1	FGF-2	Improved ependymal cell migration to injury site but no significant functional recovery	[96]
Mini-osmotic pump	Rat	Hemisection	T10	GDNF	Increased number of myelinated axons, and blood vessels, increases in axon caliber as well as a number of regenerated axons	[97]
Mini-osmotic pump	Rat	Dorsal hemisection	C2	BDNF	Significant recovery of ipsilateral hemidiaphragm EMG activity	[98]
Mini-osmotic pump	Rat	Bilateral partial transection	C5 or L2	NT-3	Reduces collateral sprouting, but enhanced CGRP axonal growth	[99]
Mini-osmotic pump	Rat	Hemisection	L1	BDNF	Enhanced connectivity of the peripheral motor bridge in a rodent model of SCI	[100]
Mini-osmotic pump + viral vectors	Rat	Dorsolateral transection	C4	BDNF	Reduced rubrospinal neuronal atrophy, and increased inflammatory damage and ion associated with mini-osmotic pump delivery	[101]
Fibrin glue	Rat	Complete transection	T8	FGF-2	No improvement in axonal myelination and functional recovery	[102]
Fibrin glue	Rat	Complete transection	T8	FGF-1	Decreased macrophage infiltration and glial cell activity, better functional recovery	[103]
Fibrin glue	Human	—	Cervical SCI	FGF-1	Improvement in ASIA grade and neurological level in most patients	[104]
Fibrin glue	Rat	Complete transection	T9	NT-3	Enhanced axonal sprouting into the lesion, reduced scarring, but no significant function improvement	[105]
Fibrin/heparin-binding system	Rat	Dorsal transection	C1, T9	NT-3, PDGF	Significantly prohibited neuronal atrophy	[106]
Fibrin	Canine	Hemisection	T10	NT-3	Inhibited inflammation, enhanced nerve fiber regeneration, and improved motor function	[107]
Hydrogel + microspheres	Rat	Hemisection	T9/10	GDNF	Improved neurite outgrowth around the lesion and functional recovery	[108]
Hyaluronan-methylcellulose hydrogel	Rat	Clip compression	T10	BDNF	Significant reduction in proinflammatory cytokines expression and cystic cavitation decreased glial scar formation, and improved neuronal survival	[109]
Hydrogel	Rat	Clip compression	T7/8	BDNF	Considerable axon preservation and astrogliosis reduction at 6 weeks post-injury without showing any inflammatory reaction, no significant improvement in the BBB score	[110]
Hydrogel with dental pulp stem cells	Rat	Clip compression	T9	FGF-2	Attenuated tissue inflammation of the injured spinal cord, resulting in a superior nerve repair	[111]
Heparin-poloxamer hydrogel	Rat	Contusion	T9/10	FGF-2, NGF	Improved neuronal survival, axon regeneration, reactive astrogliosis suppression, and locomotor recovery	[112]
Hydrogel	Rat	Hemisection	T8	NT-3	Improved axonal growth, and significant functional improvement	[113]
Hydrogel	Rat	Clip compression	T9	FGF-2	Improved functional recovery, and tissue regeneration after SCI	[114]
Hydrogel	Mice	Compression	T9	HGF	Better tissue sparing, axonal regeneration and functional recovery	[115]
Gelfoam	Rat	Contusion	C5	BDNF	Improved axonal regeneration but no significant functional recovery	[116]
Gelfoam	Rat	Complete transection	T11/12	FGF-2	Significant neural fiber increase and functional improvement	[117]
Macroporous PLA scaffold	Rat	Complete transection	T8/9	BDNF	Improved cell survival and angiogenesis but low axonal regeneration	[118]
PLGA/DC-Chol nanospheres	Rat	Hemisection	T9	VEGF	Enhanced tissue regeneration, angiogenesis, and functional recovery	[119]
PLGA + HMPCs + BMSC	Rat	Contusion	T10	FGF-2	Increased survival of implanted cells, improved functional recovery	[120]
PCL scaffold + NSCs	Rat	Dorso-ventral hemisection	T7-8	BDNF, NT-3	Better survival, migration, and neuronal and oligodendrocyte differentiation of NSCs. Increased remyelination and functional recovery	[49]
PLGA + nanofibers	Rat	Dorsal hemisection	T10	BDNF	Significantly improved functional locomotor recovery, reduced cavity formation, and increased the number of neurons at the injury site	[121]
Alginate poly-L-ornithine + fibroblasts	Rat	Hemisection	C4	BDNF	Significantly enhanced axonal regeneration and functional improvement	[122]
Agarose containing microtubules	Rat	Dorsal hemisection	T10	BDNF	Improved neurite growth and reduced inflammatory responses	[123]
Collagen scaffold + NSPC	Rat	Complete transection	T8-T9	BDNF	Retrograde tracing found possible axon regeneration after treatment	[124]
Gelatin sponge scaffold + BMSCs	Rat and canine	Complete transection	T10	NT-3	Cavity areas in the injury/graft site were significantly reduced due to tissue regeneration and axonal extensions	[125]
Chitosan	Rat	Complete transection	T7-8	NT-3	Improved neural regeneration, motor- and somatosensory-evoked potentials, and hind limb movement	[126]
Acellular spinal cord scaffold	Rat	Hemisection	T10	NT-3 + VEGF	Positive effects on anti-inflammation, axonal outgrowth, and locomotor recovery	[127]

Abbreviations: BDNF: brain-derived neurotrophic factor; FGF: fibroblast growth factor; GDNF: glial cell-derived neurotrophic factor; NT-3: neurotrophin-3; PDGF: platelet-derived growth factor; HGF: hepatocyte growth factor; VEGF: vascular endothelial growth factor.

### 3.1 Chitosan

Chitosan has fine biodegradability and biocompatibility, fine mechanical strength, and plasticity. Its shape, mechanical properties, and degradability can be easily controlled, and it induces little host immune reaction after transplantation. Therefore, it is widely used in wound dressing, drug delivery, and various issue engineering scaffolds, and is an excellent candidate to be used as a neural scaffold in the treatment of SCI [17,22,128].

### 3.2 Collagen

Collagen is the main component of the extracellular matrix. It has fine biocompatibility and degradability. Its collagen-binding domain (CBD) and ordered structure provide a satisfactory basis for containing the neurotrophic factors in a certain area and guiding the orderly growth of nerve axons in a certain direction [129]. Yang et al. injected collagen hydrogel imbedded with neural growth-promoting molecules to treat mouse SCI and achieved satisfactory axonal regeneration and locomotion recovery [130].

### 3.3 Fibrin glue

Fibronectin is a glycoprotein on the cell surface in blood, bodily fluids, and connective tissues. Functional fibronectin biomaterials are clustered structures that can be used to release the desired neurotrophic factors into the nervous system in a controlled manner [131,132]. In previous studies, fibronectin has been used as a carrier of soluble factors such as NGF and NT-3. Phillips et al. [133] showed that fibronectin can support the growth of fibroblasts, Schwann cells, and astrocytes, and provide an appropriate environment for axon growth in rats with SCI. Alovskaya et al. [134] used fibronectin as a scaffold for repairing SCI *in vivo*, and the results showed that the axonal growth in the scaffold was accompanied by the migration of Schwann cells and reactive astrocytes, which led to effective myelination of the regenerated axons and between functional recovery.

### 3.4 Alginate hydrogel

Alginate is a natural polysaccharide carbohydrate, which can be degraded *in vivo*, and provides three-dimensional scaffolds needed for cell growth. Alginate is cross-linked to form calcium alginate gel, which is permeable to required nutrients. After hydrolysis, products are excreted via kidneys with little toxicity [135]. Stokols and Tuszynski used alginate hydrogel to bridge tissue deficit after SCI and found that capillary tunnels in the alginate hydrogel provided ideal conditions for nerval and vascular regeneration in the injured spinal cord [136].

### 3.5 Polyglycolic acid

Polyglycolic acid is a biodegradable synthetic polymer that can be used to synthesize poly (lactic acid glycolic acid) copolymers. Polyglycolic acid carrying supportive stem cells and neurotrophic factors can be directly applied in the injury site, bypassing blood–spinal cord barrier that significantly decreases the efficacy of intravenous stem cell injection [137,138,139]. Novikov et al. [140,141] first used polyhydroxybutyrate scaffolds to treat SCI in mice and found that they could reduce the death of neurons after SCI. When the tubular polyhydroxybutyrate scaffolds were seeded with Schwann cells and transplanted into the injured spinal cord of mice, it was found to promote the survival and differentiation of Schwann cells, and the regeneration of spinal cord axons. Moore et al. [142] used the polyglycolic acid/poly(lactic acid) scaffolds containing Schwann cells to treat the injured spinal cord of rats. A month later, the axonal regeneration was observed to be significantly more robust than the control group.

### 3.6 Heparin-conjugated fibrin (HCF) gel

The HCF-loaded controlled-release system was reported to have certain advantages in clinical translation. The HCF gel can be used as an injectable material and is easy to operate, as well as various loading doses of neurotrophic factors can be modulated within the allowable range [143]. The HCF gel can sustainably deliver a variety of growth factors, including bFGF, NGF, and VEGF. Because various neurotrophic factors such as NGF and bFGF have a heparin-binding domain, heparin can combine with those factors, increase their stability, release them evenly and steadily, and enhance their bioavailability. The HCF gel compound neurotrophic factor, released in a controlled and sustained way, can be a safe and effective treatment option with great clinical potential. When fibrinogen is mixed with thrombin, they transform into fibrin through polymerization, cross-linked into a three-dimensional network structure, and finally, form fibrin glue. Fibrin glue is the main component of the natural extracellular matrix, biological material with low antigenicity, toxicity, and the properties of tissue adhesion prevention, and repairing tissue defects. Fibrin gel has a fine three-dimensional porous structure, good biocompatibility, and biodegradability. It is an ideal scaffold material for tissue engineering. It can not only be used as an active cell scaffold, but also as drug controlled release system for various neurotrophic factors with a short half-life cycle. Itosaka et al. [144] planted bone marrow mesenchymal stem cells (BMSCs) as seed cells on the three-dimensional scaffold constructed by fibrin and then implanted them into the SCI site of mice. They found that the fibrin scaffold significantly improved the survival of BMSCs, promoted cellular migration, and improved functional recovery after SCI. Taylor et al. [145] also reported significantly thickened heparin spinal nerve fibers after implanting the fibrin gel into the injured spinal cord.

### 3.7 Agarose

Agarose is mainly composed of galactose and its derivatives. It has a uniform structure, little toxicity, and porous structure that enables it to imbed necessary stem cells and neurotrophic factors [146]. While it is mainly used for cell culture, it can also be used as a scaffold material in tissue engineering. Stokols and Tuszynski [136] used porous freeze-dried agarose scaffold combined with recombinant BDNF to treat adult rat SCI models, and it significantly promoted axon regeneration at the injured site.

### 3.8 Synthetic polymers

Synthetic polymers have been widely used as surgical sutures for more than 20 years, and many other synthetic polymers have also been approved by food and drug administration. Synthetic materials used as tissue engineering scaffolds have many advantages: they can be designed according to the specific requirements of mechanical properties and degradation rate, can incorporate various properties into one piece of the scaffold, and have more reliable sources of raw materials [147,148].

#### 3.8.1 Synthetic hydrogels

The synthetic hydrogel has no degradability *in vivo* and can form three-dimensional scaffolds needed for cell adhesion and axonal growth. The mechanical properties of those scaffolds are very similar to those of the spinal cord. They induce little immune reaction after the implantation into the injured spinal cord, and the regenerating axons can grow into the scaffold [149]. Hejcl et al. [150] implanted synthetic hydrogels 7 days after SCI and found that the formation of spinal cord cavity was inhibited while axon regeneration and myelination were promoted, suggesting that synthetic hydrogel can inhibit the formation of glial scar after SCI, and provide a suitable microenvironment for spinal cord axonal regeneration and myelination.

#### 3.8.2 Polylactic acid

Polylactic acid is made from renewable plant resources. Polylactic acid is a biodegradable polymer material with good biocompatibility, and it can be 3-D printed into any shape that is needed at the site of injury [151]. Patist et al. [118] transplanted polylactic acid-containing brain-derived growth factors into the spinal cord of rats with thoracic cord transection. The results showed that the survival rate of tissues was significantly increased, the vascular growth was more prominent, and the axonal growth was slow, indicating that a more intense nonspecific aseptic inflammatory reaction was induced by polylactic acid.

#### 3.8.3 Polyethylene glycol (PEG)

PEG has low toxicity, fine water solubility, and good compatibility with many organic components. PEG can be used as a sealant for axonal membrane damage. Luo and Shi [152] implanted PEG at the site of SCI. The results showed that PEG can significantly inhibit the activity of apoptotic protease-3 and inhibit programmed cell mortality. They hypothesized that PEG can repair the injured cell membrane, protect the function of mitochondria, inhibit cell apoptosis, and hence, promote neural regeneration and repair after SCI.

A combined application of neurotrophic factors, stem cells, and tissue engineering scaffolds have shown positive results in providing continuous neurotrophic support to the SCI injury site, accelerating neural regeneration, and promoting functional recovery below the injury level. Chen et al. [153] reported that the acellular biological scaffolds can improve the survival of seed cells, promote their migration and differentiation, and reduce the apoptosis of host nerve cells, so as to protect the host tissue and promote functional recovery after SCI. Zhou et al. [154] used polycaprolactone scaffolds containing activated Schwann cells and NSCs derived from IPSCs to treat SCI rats. The results showed that the cells grew well on the scaffolds, and this method reduced the volume of the lesion cavity and promoted the recovery of motor function of rats. Ferrero-Gutierrez et al. [155] treated SCI rats with a novel serum-derived albumin scaffold inoculated with adipose-derived stem cells and OECs. After 45 days, it was found that the scaffold played a positive role in filling the lesion cavity and reducing the formation of the glial scar. The acellular biological scaffold inoculated with human umbilical cord blood BMSCs were also reported to bridge the spinal cord cavity and promote axonal regeneration and functional recovery in SCI rats [156]. Lin et al. [157] designed and tested a collagen scaffold according to the structural characteristics of the spinal cord. They fused the CBD with neurotrophic factors to store and release neurotrophic factors including NT3, BDNF, NGF, and bFGF in controlled fashion. When the ordered nerve regeneration collagen scaffold and neurotrophic factor with specific collagen binding ability were applied to the injured site in rat and canine SCI models, neurotrophic factors were continuously released and maintained at a certain concentration, thus reducing the injury volume and CSPGs deposition, and guiding nerve regeneration and axonal myelin formation, resulting in significantly promoted motor function recovery. The CBD-NT3 controlled release system was also used in canine and nonhuman primate models with satisfactory results by Han et al. [158,159]. The Langer team of MIT achieved using poly-l-lactic-polyglycolic acid biodegradable scaffolds to promote tissue remodeling and functional improvement in nonhuman primates with acute SCI with satisfactory results and moved into human trials [160].

## 4 When and for how long?

To promote neural regeneration after SCI, genetically modified cells, viral vectors, recombinant proteins such as osmotic pumps, biopolymers as well as natural and synthetic scaffolds were used to sustainably deliver the neurotrophic factors to the site of SCI. To prevent loss of spinal neurons, and retraction and demyelination of axons, in most studies the neurotrophic factors were delivered immediately after the injury at the lesion site. While there is less frequent application of subacute (within 2 weeks after injury) injection, there are a few reports of their application in the chronic phase. Nonetheless, there are several animal studies and [161] and human trials [162] that reported improved neural regeneration and functional recovery after weeks, months, and even up to 8.5 years after injury. There are no systematic studies comparing the time at which the neurotrophic factors should be sustained in the injury site for better morphological preservation and functional recovery [12]. Olfactory ensheathing glia overexpressing BDNF or GDNF maintained the expression of those neurotrophic factors up to 8 weeks after injection [63,65]. Adenoviral injections were reported to overexpress BDNF and NT-3 at the injury site as long as 16 weeks after injection [38]. The biomaterial PLA-bPEG-b-PLA hydrogel was reported to keep the concentration of NT-3 elevated at the injury site 2 weeks after injection [113]. Note, however, that long-term vector-mediated expression of growth factors can not only potentially entrap axons but also alter the dendritic architecture of both the transduced and adjacent nontransduced neurons [163], and can induce major factor-specific changes in the expression of endogenous genes in tissues containing transduced neurons [164]. Clearly, further studies are needed to determine the optimal concentration and duration of neurotrophic support at SCI sites.

With the rapid advancement of tissue engineering techniques and satisfactory animal experiment results, there are numerous examples of human trials involving different neurotrophic factors, stem cells, and tissue engineering scaffolds for their timely and abundant delivery. However, there are not yet any reports of recovery after total SCI. Considering the complex biomechanical changes after SCI, any single approach has proven to be unsatisfactory. Adequate combination of neurotrophic factors that can be released into the injury site in a controlled manner using cellular and viral vehicles with tissue engineering materials is necessary to achieve satisfactory functional recovery after acute SCI.
